# Case Report: donor cell-derived leukemia after allogeneic stem cell transplantation for metastatic renal cell carcinoma

**DOI:** 10.3389/fimmu.2026.1830323

**Published:** 2026-05-26

**Authors:** Federico Monaco, Danh T. Tran, Brenda M. Sandmaier, Scott S. Tykodi

**Affiliations:** 1 Azienda Ospedaliero-Universitaria SS Antonio e Biagio e Cesare Arrigo, S.C.D.U. Ematologia, Alessandria, Italy; 2 Fred Hutchinson Cancer Center, Clinical Research Division, Seattle, WA, United States; 3 Division of Hematology and Oncology, University of Washington, Seattle, WA, United States; 4 Fred Hutchinson Cancer Center, Translational Science and Therapeutics Division, Seattle, WA, United States

**Keywords:** acute myelomonocytic leukemia, allogeneic hematopoietic cell transplantation, autologous hematopoietic cell transplantation, donor cell-derived leukemia, renal cell carcinoma

## Abstract

Donor cell-derived leukemia (DCL) is a rare complication of allogeneic hematopoietic cell transplantation (allo-HCT), previously described in patients receiving allo-HCT for hematologic malignancies. Allo-HCT has also been investigated as an immunotherapy platform for solid tumors including clear cell renal cell carcinoma (RCC). Herein, we describe a unique patient case treated by allo-HCT for RCC who subsequently developed DCL. A 40-year-old man was diagnosed with metastatic clear cell RCC treated surgically by left nephrectomy plus metastasectomy of a rib lesion. He was referred to the Fred Hutchinson Cancer Center and treated by allo-HCT as part of a clinical trial (NCT00005851). His initial allograft following fludarabine/2 Gy total body irradiation (TBI) conditioning was complicated by graft failure. After collecting a G-CSF-mobilized autologous hematopoietic stem cell product as a failsafe for repeated graft failure, he was re-transplanted from the same donor following cyclophosphamide/antithymocyte globulin (ATG) conditioning, resulting in successful engraftment. Fourteen months later, he developed donor origin acute myelomonocytic leukemia with central nervous system (CNS) involvement. He was treated by 7 + 3 induction with intrathecal methotrexate and was able to achieve complete remission. He subsequently underwent autologous hematopoietic cell transplantation (auto-HCT) without further leukemia relapse. Unfortunately, 4 years after his auto-HCT, he developed recurrent RCC, initially managed by surgical resections. Upon further progression, he was then treated with sequential lines of systemic therapy that included pazopanib, nivolumab, cabozantinib, and lenvatinib/everolimus. He died from complications of his RCC 17 years after his second successful allograft. The absence of pre-transplant systemic therapies for the patient’s metastatic RCC and ongoing good health of the donor implicates a leukemogenic potential of his transplant-associated therapies including TBI, cyclophosphamide, and ATG. His uncommonly long survival from metastatic RCC suggests clinical benefit was derived from the 14-month duration of allo-HCT associated with T-cell allo-immunity previously shown capable of targeting RCC-associated minor histocompatibility antigens.

## 1 Introduction

Donor cell-derived leukemia (DCL) is a rare complication of allogeneic hematopoietic cell transplantation (allo-HCT) that was first recognized in 1971 by Fialkow (1). Since then, numerous additional cases have been described in the literature, with an estimated incidence following allo-HCT ranging from 0.08% to 0.84% as reported in several cohort studies (2).

This complication is characterized by the emergence of a leukemic clone in donor cells and the development of overt acute lymphoid or myeloid leukemia. Different methods are used to confirm the origin of the neoplastic cells, including cytogenetic, fluorescence *in situ* hybridization, and molecular DNA markers [short tandem repeats (STRs) or variable number of tandem repeats (VNTRs)] (3). Characterization of donor health status at the time of DCL diagnosis has observed a hematologic malignancy emerging in the donor in only a small fraction of cases (4).

Several hypotheses have been proposed regarding the mechanism contributing to leukemogenesis in the allo-HCT recipient: subclinical cancer predisposition in donors with inherited genetic mutation, clonal hematopoiesis with somatic gene mutation due to aging, immune dysfunction caused by allo-HCT, the transfer of an etiologic agent such as a virus or genetic material from host to donor cells, or an alteration of the bone marrow microenvironment (2).

All previously reported cases of DCL arose in patients who underwent allo-HCT in order to treat a hematological disease originating from or involving the bone marrow (2, 4–11). In this report, we present a case of donor cell-derived acute myelomonocytic leukemia (AMML) developing in an adult patient who underwent allo-HCT to treat metastatic clear cell renal cell carcinoma (RCC) without prior exposure to chemotherapy or radiotherapy.

## 2 Case description

### 2.1 Pre-transplant history

**Figure 1:**
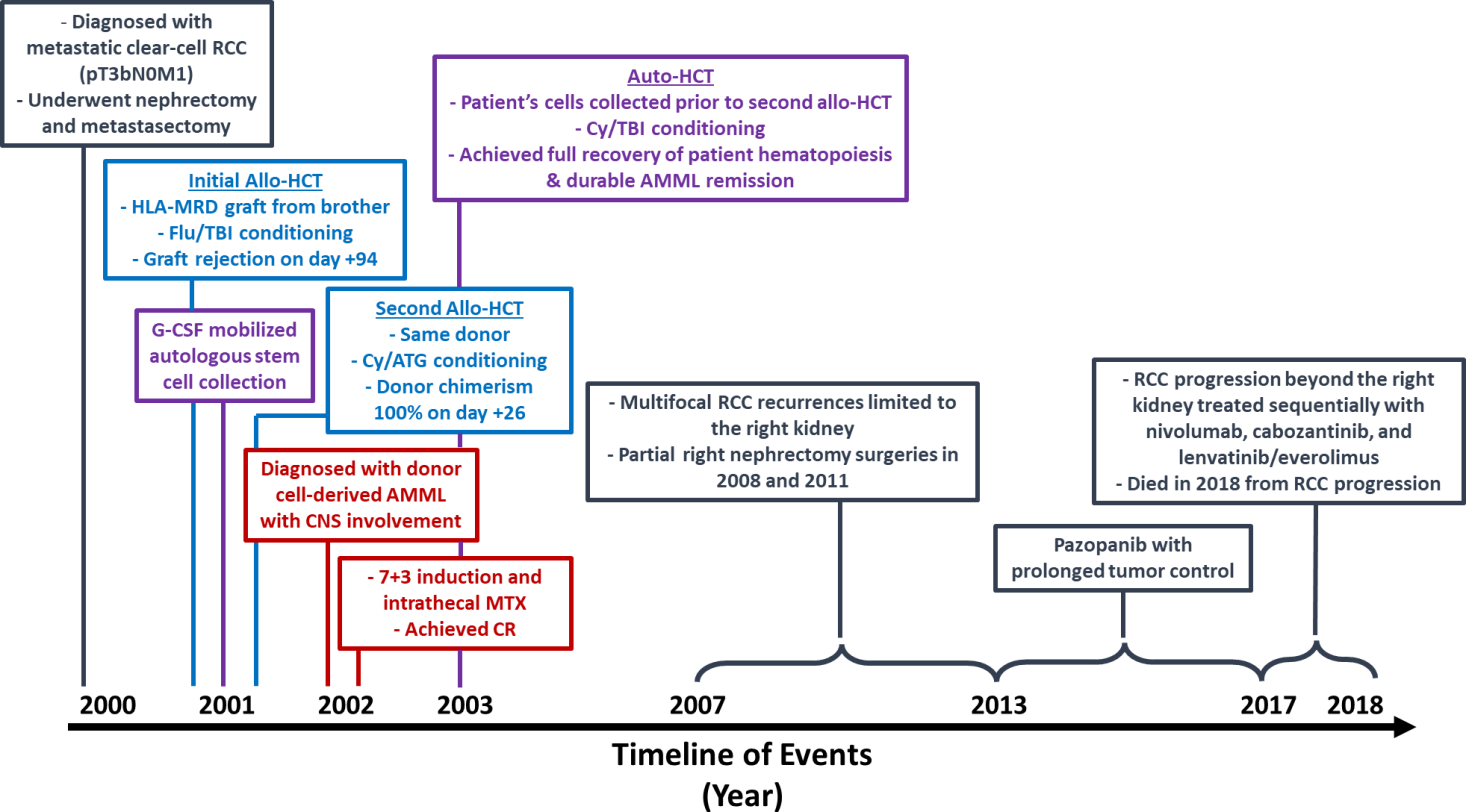
Clinical time course for a patient with metastatic renal cell carcinoma treated by allo-HCT. Allo-HCT, allogeneic hematopoietic cell transplantation; AMML, acute myelomonocytic leukemia; ATG, antithymocyte globulin; Auto-HCT, autologous hematopoietic cell transplantation; CNS, central nervous system; CR, complete remission; Cy, cyclophosphamide; Flu, fludarabine; GvHD, graft-versus-host disease; HLA-MRD, HLA-matched related donor; MTX, methotrexate; RCC, renal cell carcinoma; TBI, total body irradiation.

In September 2000, a 40-year old Asian man presented with gross hematuria with clots; computed tomography (CT) imaging showed a 16 × 13cm mass in his left kidney and a 2 × 5 cm mass in the right third rib. In September 2000, he underwent a left nephrectomy with retroperitoneal lymphadenectomy followed by a metastasectomy surgery resecting the right third rib lesion. Histological analysis from the left nephrectomy showed a 20-cm clear cell RCC, Fuhrman grade II, with negative lymph nodes. The rib metastasis had sarcomatoid histology. Pathologic staging was pT3b, N0, M1 (**Figure 1**).

No other treatment was given, and the patient was then evaluated at the Fred Hutchinson Cancer Center for enrollment in a protocol for an HLA-matched related nonmyeloablative allo-HCT as treatment for metastatic clear cell RCC. Pre-transplant patient characteristics are shown in **Table 1**. The patient was found to have an HLA-identical brother and was enrolled in the multicenter protocol (NCT00005851).

**Table 1:** Pre-transplant patient characteristics.

Clinical Variable	Data
Age:	40 years
Sex:	Male
Ethnicity:	Pacific Islander
Allergies:	None
Alcohol/Drug abuse:	None
Tobacco use:	17 pack-years of cigarettes; discontinued 6 years prior to transplant
Past medical history:	Appendectomy in 1984
Family history:	No first-degree relatives with cancer history. The patient declined genetic testing for a germline genetic association with his RCC.
Sibling donor:	HLA-identical brother alive without medical issues
Occupational exposure:	None known
Serologies:	Negative for past infection with HSV, CMV, Toxoplasma; positive for VZV

CMV, cytomegalovirus; HLA, human leukocyte antigens; HSV, herpes simplex virus; RCC, renal cell carcinoma; VZV, varicella zoster virus.

### 2.2 Initial Allo-HCT

The patient started conditioning in April 2001 with the following regimen: fludarabine 30 mg/m^2^ on days −4, −3, and −2 and total body irradiation (TBI) using 2 Gy in a single session on day 0. The patient then received an unmodified G-CSF mobilized peripheral blood stem cell graft containing 13.23 × 10^6^ CD34^+^ cells/kg. The apheresed donor product had been cryopreserved before infusion to accommodate the donor’s military service schedule. Graft-versus-host disease (GvHD) prophylaxis treatment included mycophenolate mofetil (MMF) 15 mg/kg PO q8h on days 0–40 and cyclosporine 5.0 mg/kg PO q12h on days –3 to +35 then tapering to day +56. Neutropenia lasted 7 days with prompt hematopoietic recovery. Chimerism analysis was based on two informative genomic loci, ApoB, a VNTR, and SE-33, a large STR, with an estimated detection sensitivity of 1%–5%. The patient was found to have mixed chimerism on day +28 evaluation: peripheral CD3^+^ cells were 40%–50% donor and CD33^+^ cells were 50%–60% donor; unsorted bone marrow analysis showed 50%–60% cells of donor origin. On day +56, the patient developed fever due to a bacterial infection, and subsequently, his absolute neutrophil count started to fall without response to G-CSF. On day +73, a bone marrow biopsy was done with findings concerning for graft failure (minimal trilineage hematopoiesis, chimerism of peripheral CD3^+^ cells was 20%–30% donor, and unsorted bone marrow 5%–10% donor). Repeated chimerism analysis on day +94 demonstrated 100% host hematopoiesis confirming rejection of the donor graft and recovery of autologous hematopoiesis (**Figure 1**).

### 2.3 Second allo-HCT

The patient was offered a second attempt at allo-HCT from the same donor with an immunosuppressive conditioning regimen that was standard for aplastic anemia. As a safety strategy, prior to initiating conditioning for his second all-HCT, the patient underwent G-CSF mobilization with apheresis collection of an unmodified autologous stem cell product containing 1.37 × 10^6^ CD34^+^ cells/kg to be available for an autologous HCT (auto-HCT) in the event of a second graft rejection and marrow aplasia.

The patient started conditioning in August 2001 with the following regimen: cyclophosphamide 50 mg/kg on days −5, −4, −3, and −2 and anti-thymocyte globulin (ATG) 30 mg/kg on days −4, −3, and −2. Subsequently, the patient received a fresh unmodified G-CSF mobilized peripheral blood stem cell graft containing 10.3 × 10^6^ CD34^+^ cells/kg. GvHD prophylaxis treatment included MMF 15 mg/kg PO q8h on days 0–27 and cyclosporine 5.0 mg/kg PO q12h on days –3 to +56 then tapering. Neutropenia lasted 14 days without infectious complications. Chimerism analysis and bone marrow evaluation showed a 100% donor chimerism on transplant day +26, and all subsequent assessments throughout the first year (days +56, +84, +180, and +365). No GvHD developed during immunosuppressive drug tapering and GvHD prophylaxis was concluded at +245 days (**Figure 1**).

### 2.4 Donor cell-derived acute myelomonocytic leukemia

In November 2002, day +440 following the second allo-HCT, the patient underwent peripheral blood analysis with new evidence of leukopenia. At the same time, he reported fever, skin lesions, and nosebleeds. Subsequently, the patient rapidly developed pancytopenia. A bone marrow biopsy revealed that, morphologically, blasts and promonocytes comprised 81% of marrow cellularity. Cytochemical studies demonstrating positivity of blasts for both MPO and NSE stains on greater than 20% of the marrow cells favored classification as leukemia AMML according to the World Health Organization 2001 classification of the myeloid neoplasms (12). Cytogenetics showed a male karyotype without abnormalities (46, XY). A lumbar puncture was performed with evidence of low-level central nervous system (CNS) involvement by AMML. Chimerism analysis of peripheral blood and marrow cells showed 100% donor, and a chimerism analysis of flow-sorted blasts also showed 95%–99% donor origin. Taken together, the findings confirmed a diagnosis of donor cell-derived AMML.

Induction therapy was started according to a 7 + 3 scheme (cytarabine 200 mg/m^2^ for 7 days, daunorubicin 90 mg/m^2^ for 3 days). No major complications occurred during the aplastic phase. Post-induction bone marrow evaluation showed complete remission with full-donor chimerism. Two prophylactic intrathecal infusions of methotrexate were done in consideration of the low-level cerebrospinal fluid involvement (**Figure 1**).

### 2.5 Auto-HCT

To provide definitive allogenic immunotherapy for the donor-origin AMML, the patient received an auto-HCT in February 2003 using his autologous stem cells mobilized before the second allo-HCT. Conditioning involved cyclophosphamide 60 mg/m^2^ and TBI 12 Gy. His duration of severe neutropenia lasted for approximately 21 days. The clinical course was complicated by severe respiratory syncytial virus (RSV) pneumonia with respiratory failure needing therapy with ribavirin plus palivizumab and also intubation with mechanical ventilation. Interestingly, the patient developed acute GvHD 18 days after auto-HCT (574 days after his second allo-HCT) localized to skin and confirmed by punch biopsies. Furthermore, 30 days after auto-HCT, he also developed acute gastric GvHD, again confirmed with biopsy; each presentation rapidly disappeared after a short course of steroids.

Post-transplant bone marrow evaluation confirmed complete remission from AMML and recovery of host hematopoiesis with 100% autologous chimerism. Four additional prophylactic intrathecal methotrexate infusions were done in the subsequent months (**Figure 1**).

### 2.6 Post-transplant follow-up

No signs of leukemia were seen at successive bone marrow examinations at days +30 and +74 after auto-HCT. In November 2003, the patient developed *Pneumocystis* pneumonia treated with trimethoprim/sulfamethoxazole. In January 2004, streptococcal pneumonia was diagnosed requiring broad-spectrum antibiotics. In February 2005, the patient presented with an upper respiratory infection and interstitial bronchitis with culture samples positive for *Streptococci* and *Haemophilus influenzae* requiring antibiotics. Because of recurrent respiratory infections and hypogammaglobulinemia, the patient was given I.V. immunoglobulin prophylaxis from 2005 to 2007.

### 2.7 RCC progression

In February 2007, two new masses measuring up to 1.9cm were seen in the right kidney, and the patient underwent open partial nephrectomy in 2008 for excision of these two masses. In late 2011, multifocal recurrence in the right kidney was again observed, and he subsequently underwent another open partial nephrectomy for excision of three masses. Pathology from these surgeries showed clear cell RCC for all tumors. Because of further RCC progression in the right kidney, the patient opted for management by systemic medical therapy in preference to radical nephrectomy that would result in an anephric status requiring hemodialysis. Beginning in May 2013, the patient was treated with pazopanib, an antiangiogenic tyrosine kinase inhibitor, and achieved a long interval of disease control lasting until August 2017. Upon RCC progression, the patient’s systemic therapy was then transitioned to nivolumab, but by December 2017, he experienced RCC progression beyond the right kidney with new metastatic sites including lung, lymph nodes, and peritoneum. He received additional lines of therapy with cabozantinib, followed by lenvatinib plus everolimus. Unfortunately, the patient died in September 2018 from progressing RCC further complicated by a hepatic abscess at the site of a necrotic metastatic tumor. There were no signs of leukemia relapse (15 years after autologous transplantation). At the time of the patient’s death, his brother was alive, healthy, and without evidence of hematologic malignancy (**Figure 1**).

## 3 Discussion

The mechanism of leukemogenesis in DCL remains uncertain, but is likely multifactorial with several processes having been proposed, including donor-related factors (such as occult malignant clones in the donor, acquired genetic predisposition with age or germline mutation), recipient bone marrow microenvironment factors, leukemogenic transplant conditioning regimens, and post-transplant immunosuppressed state (2, 13).

In this report, we describe a patient who developed donor cell-derived AMML after undergoing allo-HCT for metastatic RCC. The underlying solid tumor indication for allo-HCT was noteworthy as the patient had received no disease-specific chemotherapy prior to transplant, which stands in marked contrast to patients with hematologic cancers. However, a number of the patient’s treatments over the course of his three sequential HCTs have been associated with the appearance of DCL.

The role of conditioning was investigated in previous reviews of donor cell-derived leukemia. TBI has been associated with a significant increase in overall cancer risk following allo-HCT (14) and is a risk factor for DCL. In a Japanese review (15), 30 of 40 patients with donor cell-derived leukemia underwent TBI, and in a European review (16), 7 of 14 patients were exposed to radiotherapy (6 TBI and 1 TLI). Wiseman (13), in his detailed review of DCL, reported that in 54% of cases of donor cell-derived leukemia analyzed, TBI was used. It could be hypothesized that irradiation damage to the marrow microenvironment could have contributed to the development of leukemia in our patient, as the donor cells were not directly exposed to the TBI administered as part of the conditioning for his first allo-HCT. However, a dose proportional association for radiation exposure with bone marrow stromal injury and the very low dose TBI administered to this patient make a contribution for TBI in this case less certain (17). An analogous observation can be made regarding the use of cyclophosphamide as conditioning therapy. Cyclophosphamide conditioning was used in 14 of 14 patients’ conditioning in the EBMT review and in 85% of cases in the Wiseman’s review (44% of cases in combination with TBI). In addition to an indirect effect on the marrow microenvironment, our patient received cyclophosphamide conditioning for his second allo-HCT following the initial donor graft failure. It can be hypothesized that donor microchimerism was still present during the second conditioning regimen and that the remaining donor cells were exposed directly to cyclophosphamide with a resulting mutational event. The use of ATG as part of transplant conditioning has also been shown to increase risk for DCL (18), presumably as a consequence of *in vivo* T-cell depletion resulting in impaired immunosurveillance. Therefore, this agent as part of our patient’s second auto-HCT conditioning regimen could be a contributing factor leading to his AMML development.

Although identifying which factor played a predominant role in leukemogenesis is not possible, an alternative, and not mutually exclusive interpretation is a sequential multi-step/multi-hit model, in which an early mutagenic insult during the initial conditioning and engraftment period led to the emergence of pre-leukemic clones. These clones may have subsequently expanded under proliferative stimuli such as G-CSF and were further shaped by immunologic selection and/or additional genomic events acquired during the second conditioning regimen, ultimately giving rise to overt leukemia. Consistent with a multi-hit model, deletion of the long arm of chromosome 20 following allogenic HCT has recently been proposed to represent a first-hit mutation and a risk factor for the subsequent emergence of post-transplant myeloid malignancies (11).

Regarding other potential risk factors, viral involvement in leukemogenesis in our patient was judged less likely, given that virus serologies before transplantation were mostly negative (except VZV), and during the conditioning and post-transplant phase, no CMV or EBV reactivations were detected. Additionally, given his brother was alive and well throughout his 18-year clinical course, donor-derived factors such as germline mutations were unlikely.

It is noteworthy that our patient developed clinical and pathologic manifestations of low-grade cutaneous and gastric acute GvHD following his auto-HCT. An allogeneic immune reaction causing his GvHD would require a state of persistent microchimerism with tissue-resident donor-derived target cells in the skin and gut mucosa. An analogous persistence of donor-derived cells below the threshold for chimerism detection in peripheral blood is hypothesized to represent the substrate for leukemic transformation and the emergence of his donor cell-derived leukemia. The onset of GvHD 574 days after the second allo-HCT indicates a prolonged duration of microchimerism. However, similar clinical and pathologic findings can also be observed following autologous HCT, with skin and gastrointestinal tract being the most commonly involved sites (19). Therefore, a fully autologous syndrome potentially related to thymic dysfunction impairing negative selection or depletion of regulatory T cells contributing to the emergence of autoreactive T-cell clones cannot be excluded (20). Finally, while engraftment syndrome could also mimic GvHD after auto-HCT (21), the clinical timeline for gastrointestinal symptoms as well as skin and gastric biopsy findings in this case would argue against that diagnosis.

It is remarkable that our patient survived his metastatic RCC 18 years after his diagnosis with a disease that in 2000 had a median survival of approximately 1 year for patients receiving standard interferon therapy (22). It is possible that the T-cell allo-immunity present during the 14 months he maintained his donor’s allograft contributed to his survival. We previously demonstrated donor-derived, minor histocompatibility antigen-specific T cells isolated from this patient that could recognize a target antigen on RCC tumor cell lines *in vitro* (23). It is formally possible that the allograft eliminated a micro-metastatic disease burden from his original left-sided RCC tumor and the subsequent recurrence in the right kidney was a *de novo* event. Validated molecular tools to establish a clonal relationship between separate RCC tumors are not readily available.

## Data Availability

De-identified clinical data supporting the assessment of this case and the conclusions of this report will be made available by the corresponding author/s upon request.
